# Rational Design and Synthesis of Altered Peptide Ligands based on Human Myelin Oligodendrocyte Glycoprotein 35–55 Epitope: Inhibition of Chronic Experimental Autoimmune Encephalomyelitis in Mice

**DOI:** 10.3390/molecules191117968

**Published:** 2014-11-04

**Authors:** Theodore Tselios, Mihalis Aggelidakis, Anthi Tapeinou, Vivian Tseveleki, Ioannis Kanistras, Dimitrios Gatos, John Matsoukas

**Affiliations:** 1 Department of Chemistry, University of Patras, 26500 Patras, Greece; E-Mails: mihalis_ali@hotmail.com (M.A.); atapeinou@upatras.gr (A.T.); d.gatos@upatras.gr (D.G.); 2 Department of Molecular Genetics, Hellenic Pasteur Institute, 11521 Athens, Greece; E-Mails: vtseveleki@pasteur.gr (V.T.); ikanistras@pasteur.gr (I.K.)

**Keywords:** multiple sclerosis, experimental autoimmune encephalomyelitis, altered peptide ligand, cyclic peptides

## Abstract

Experimental autoimmune encephalomyelitis (EAE) is a demyelinating disease of the central nervous system and is an animal model of multiple sclerosis (MS). Although the etiology of MS remains unclear, there is evidence T-cell recognition of immunodominant epitopes of myelin proteins, such as the 35–55 epitope of myelin oligodendrocyte glycoprotein (MOG), plays a pathogenic role in the induction of chronic EAE. Cyclization of peptides is of great interest since the limited stability of linear peptides restricts their potential use as therapeutic agents. Herein, we have designed and synthesized a number of linear and cyclic peptides by mutating crucial T cell receptor (TCR) contact residues of the human MOG_35–55_ epitope. In particular, we have designed and synthesized cyclic altered peptide ligands (APLs) by mutating Arg^41^ with Ala or Arg^41^ and Arg^46^ with Ala. The peptides were synthesized in solid phase on 2-chlorotrityl chloride resin (CLTR-Cl) using the Fmoc/*t*-Bu methodology. The purity of final products was verified by RP-HPLC and their identification was achieved by ESI-MS. It was found that the substitutions of Arg at positions 41 and 46 with Ala results in peptide analogues that reduce the severity of MOG-induced EAE clinical symptoms in C57BL/6 mice when co-administered with mouse MOG_35–55_ peptide at the time of immunization.

## 1. Introduction

Multiple sclerosis (MS) is a chronic and inflammatory disease of the central nervous system (CNS) characterized by demyelination and loss of neurologic function [1,2,3,4]. MS is generally considered to be an autoimmune disease in which a coordinated attack of the immune system against components of the myelin sheath occurs [5]. Experimental autoimmune encephalomyelitis (EAE), a CD4^+^ T cell-mediated disease, is one of the best studied experimental animal models of MS, and represents an invaluable *in vivo* system for the evaluation of new therapeutic approaches [6,7]. Candidate autoantigens include constituents of the myelin sheath such as myelin basic protein (MBP), proteolipid protein (PLP) and myelin oligodendrocyte glycoprotein (MOG). New approaches in applied research for MS include the design and synthesis of peptide analogues of myelin proteins to induce tolerance against the induction of disease [8,9,10,11,12,13,14,15,16,17,18]. MOG is a transmembrane glycoprotein of the CNS and is located on the external surface of the myelin sheath surrounding the neurons. MOG is thought to be one of the autoantigens involved in MS pathology in humans [19,20,21,22,23]. The main immunodominant region of the MOG protein is the 35–55 epitope, therefore may be a primary target for the stimulation of autoreactive T-cells, in autoimmune diseases of the CNS [24,25,26,27]. The immunodominant epitopes of myelin proteins may be useful for the design and synthesis of mutated peptide analogues (altered peptide ligands, APLs) in the research for the inhibition of EAE.

The backbone cyclization of peptides has been demonstrated that the *in vivo* stability is increased and the conformational freedom is reduced. More specifically, cyclic peptides have: (i) greater bioavailability and higher resistance to proteolytic degradation, making them better potential candidates for therapeutic molecules and (ii) more precise conformation and increased receptor selectivity and specificity. The lack of conformational flexibility, which is an important characteristic of linear counterparts, confirms or eliminates their bioactive conformation [28,29,30,31].

In this work, we pursued, for the first time, the development of an antagonist peptide based on MOG_35–55_ epitope and also the development of cyclic peptide analogues and investigated their possible inhibitory effects in the induction of chronic EAE in mice. However, the therapeutic utilization of linear peptides is limited because of their degradation in the presence of proteolytic enzymes [10,13,14,16]. In an effort to develop more stable peptide analogues, without compromising their biological activity, it is necessary to pursue the design of cyclic peptides that are more resistant to proteolytic hydrolysis.

In this study, we have designed, synthesized and evaluated for their *in vivo* biological activity in EAE suppression of clinical symptoms, linear and cyclic peptide analogues of MOG_35–55_ epitope (based on the human MOG sequence: (MetGluValGlyTrpTyrArg^41^Pro^42^ProPheSerArg^46^ValValHisLeuTyrArgAsnGlyLys) with substitutions in amino acids that interact with the TCR and perhaps play an important role in EAE induction. Human MOG_35–55_ epitope differs from rat MOG_35–55_ at residue 42, including a Pro instead of Ser. The human MOG_35–55_ is weakly encephalitogenic compared to rat MOG_35–55_ in C57BL/6 mice because EAE can be induced by two mechanisms either via an encephalitogenic T cell response to rat MOG_35–55_ epitope or by an encephalitogenic B cell response to human MOG_35–55_ epitope [32]. The synthesis of linear and cyclic peptides were accomplished on CLTR-Cl resin following the Fmoc/*t*-Bu synthetic methodology.

## 2. Results and Discussion

**Scheme 1:**
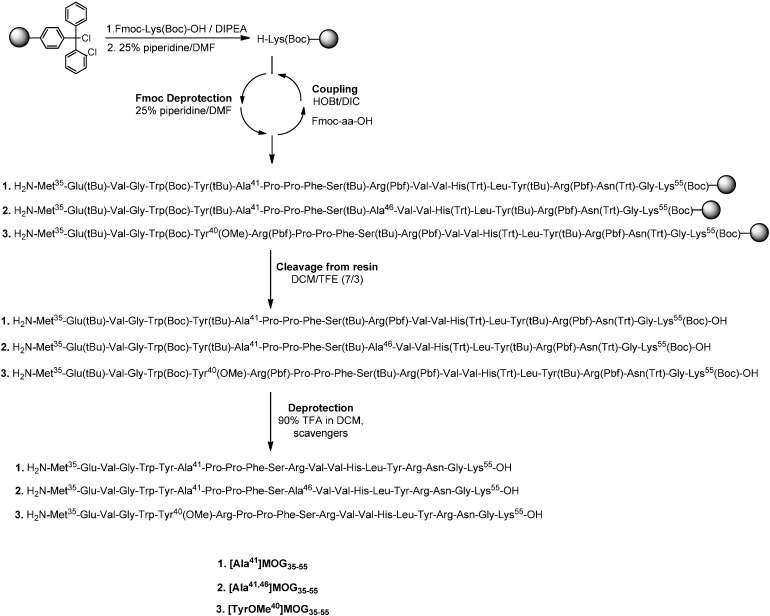
Solid Phase Peptide Synthesis of the linear mutated analogues of MOG_35–55_.

**Scheme 2:**
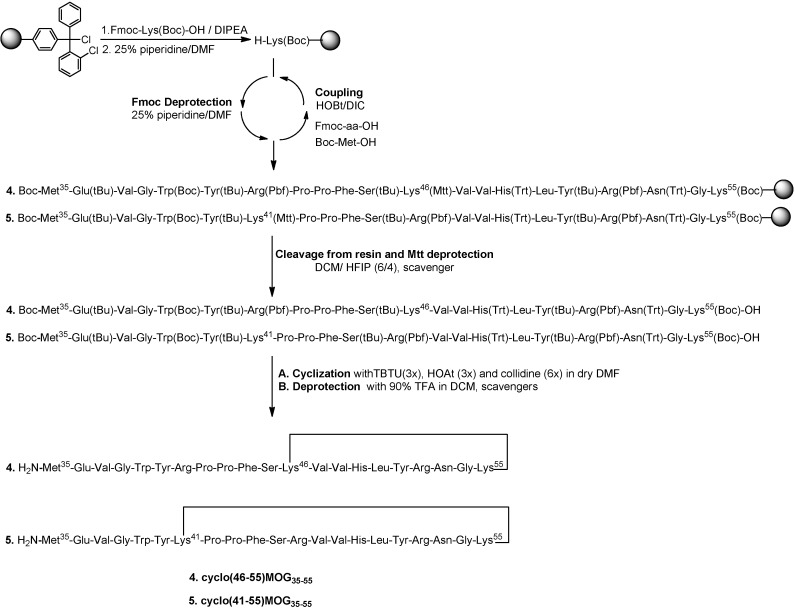
Procedure for the synthesis of the cyclic mutated analogues of MOG_35–55_.

**Figure 1:**
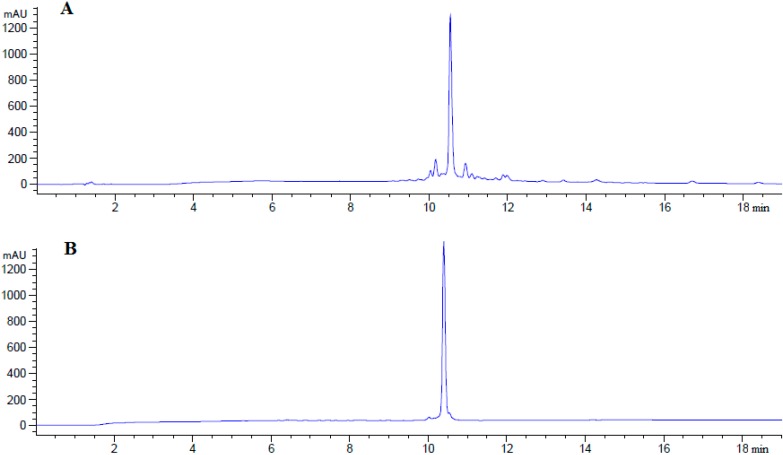

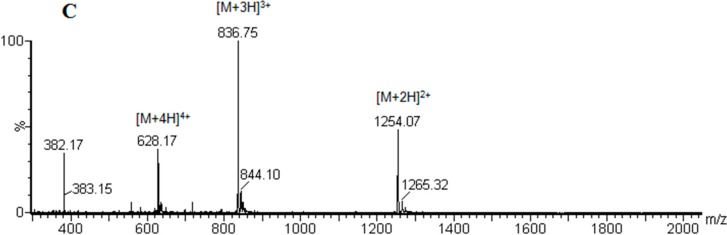
Analytical RP-HPLC of crude (**A**) and purified (**B**) [Ala^41^]MOG_35–55_ analogue; identification of synthesized analogue using ESI-MS (**C**).

**Figure 2:**
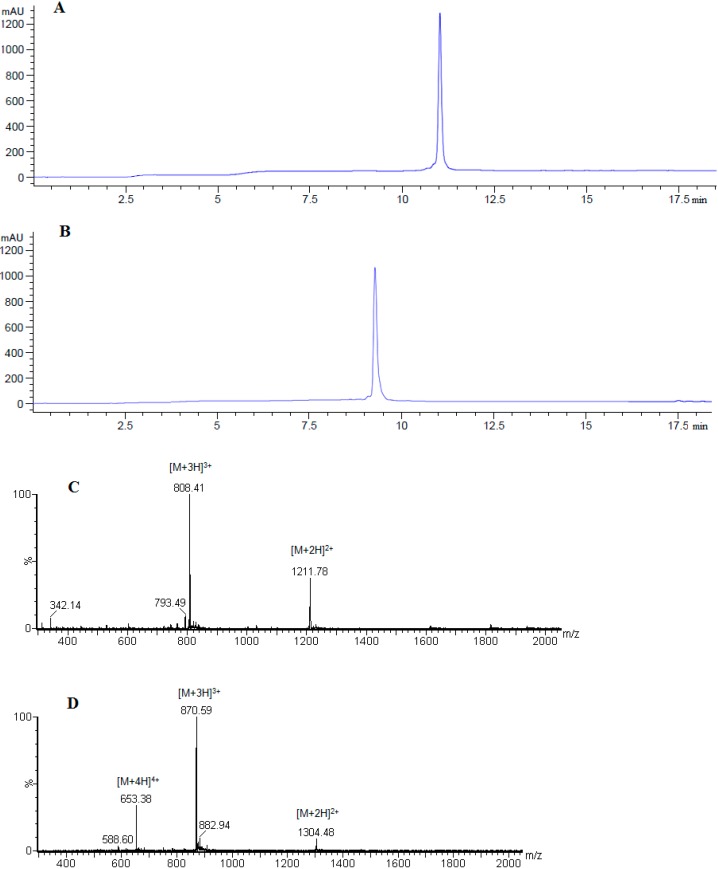
Analytical RP-HPLC of purified linear [Ala^41,46^]MOG_35–55_ (**A**) and [TyrOMe^40^]MOG_35–55_ (**B**) analogues; identification of synthesized linear [Ala^41,46^]MOG_35–55_ (**C**) and [TyrOMe^40^]MOG_35–55_ (**D**) analogues using ESI-MS.

**Figure 3:**
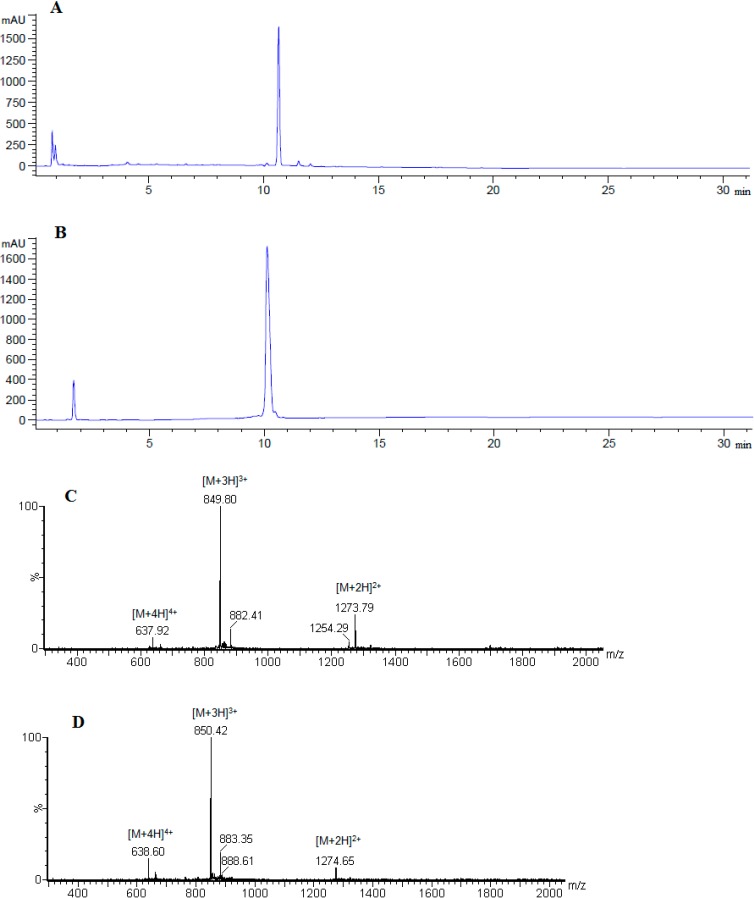
Analytical RP-HPLC of purified cyclo(46–55)MOG_35–55_ (**A**) and cyclo(41–55)MOG_35–55_ (**B**) analogues; identification of synthesized cyclo(46–55)MOG_35–55_ (**C**) and cyclo(41–55)MOG_35–55_ (**D**) analogues using ESI-MS.

**Figure 4:**
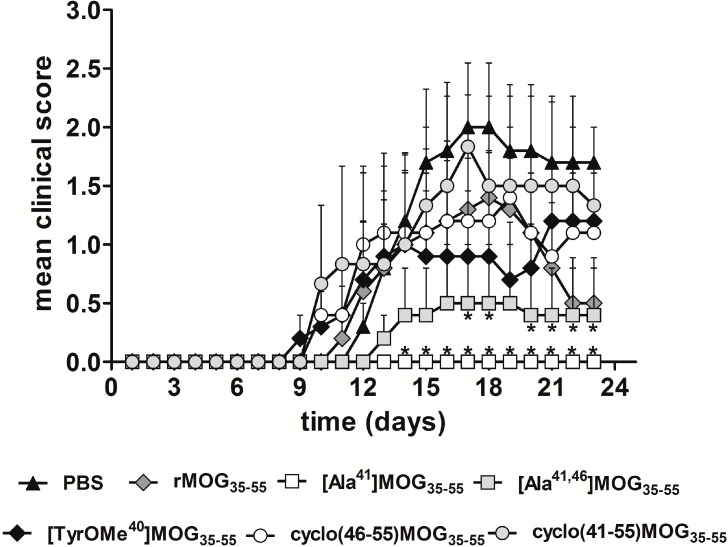
Co-administration of linear APLs, [Ala^41,46^]MOG_35–55_ and [Ala^41^]MOG_35–55_, ameliorate the clinical symptoms of rat MOG_35–55_-induced EAE in C57BL/6 mice. Mean clinical scores of MOG-EAE are shown for all groups of mice against time (*n* = 3 for the cyclo (41–55) MOG_35–55_ and *n* = 5 for other groups). Statistical significance after pair-wise comparisons (using Student’s *t* test) of each experimental group with the non-vaccinated control (PBS) group is shown (* *p* < 0.05).

Scheme 1 shows the synthesis of linear peptide analogues [Ala^41^]MOG_35–55_, [Ala^41,46^]MOG_35–55_ and [TyrOMe^40^]MOG_35–55_ by Fmoc/tBu methodology using the 2-chlorotrityl chloride resin. Scheme 2 shows the synthesis of cyclic analogues cyclo(46–55)MOG_35–55_ and cyclo(41–55)MOG_35–55_. Figure 1, Figure 2 and Figure 3 show the spectra of high performance liquid chromatography (HPLC) and electron spray ionization mass spectrometry (ESI-MS). Figure 4 shows the ability of synthesized compounds to inhibit the EAE induced by the encephalitogenic MOG_35–55_. The linear [Ala^41^]MOG_35–55_ prevents the development of disease while the cyclic analogues supress the disease in a lower degree compared to linear counterparts. Table 1 shows analytical data of synthesized compounds.

**Table 1:** Analytical data of the synthesized linear and cyclic peptides.

No.	Peptide	t_R_ (min)	HPLC Purity % ^#^	HPLC Purity % ^≠^	M_calc._ (Da)
**1**	[Ala^41^]MOG_35–55_	10.23	75.3	98.2	2505.27
**2**	[Ala^41,46^]MOG_35–55_	10.89	74.3	98.9	2420.21
**3**	[TyrOMe^40^]MOG_35–55_	09.16	58.2	98.7	2603.35
**4**	cyclo(46–55)MOG_35–55_	10.57	29.1	97.7	2545.97
**5**	cyclo(41–55)MOG_35–55_	09.96	31.8	97.9	2545.97

**^#^** It is referred to crude (unpurified) peptides, after the final deprotection, based on the HPLC chromatograms; **^≠^** It is referred to purified peptides, used in EAE experiments, based on the HPLC chromatograms.

### 2.1. Chemistry

The synthesis of linear peptides [Ala^41^]MOG_35–55_, [Ala^41,46^]MOG_35–55_ and [TyrOMe^40^]MOG_35–55_ as well as of the cyclic analogues cyclo(46–55)MOG_35–55_ and cyclo(41–55)MOG_35–55_ was carried out by using the Fmoc/tBu methodology, utilizing the 2-chlorotrityl chloride resin [33,34,35,36] that was previously used for the synthesis of novel cyclic amide-linked analogues of peptides based on the MBP protein [10,30,31]. The side chain of amino acids were protected as follows: Trt for Asn, His, Pbf for Arg, tBu for Tyr (or TyrOMe), Ser, Glu and Boc for Lys, Trp. In the case of cyclic analogues, in which the side chain amine group of Lys at position 41 or 46 and *C*-terminal carboxyl group formed an amide bond, we used Mtt in linear precursor peptide to protect Lys. This group was removed upon treatment of protected peptide-resin with the splitting mixture dichloromethane (DCM)/1,1,1,3,3,3-hexafluoro-2-propanol (HFIP) (6/4) that used to cleave the peptide from the resin. The use of the 2-chlorotrityl chloride resin, as well as of mild cleaving conditions, allowed the protected peptides to release from the resin and the subsequent cyclization of the desired protected peptides. Cyclization was achieved using *O*-(benzotriazol-1-yl)-*N*,*N*,*N*',*N*'-tetramethyluronium tetrafluoroborate (TBTU), 1-hydroxy-7-azabenzotriazole (HOAt) and 2,4,6-collidine (2,4,6-trimethylpyridine) allowing fast reaction and high yield cyclization product. The final deprotection of each peptide was achieved using trifluoroacetic acid (TFA) in DCM in presence of scavengers. The oxidation of thioether of Methionine to sulfoxide was revealed as a small peak, just before the main peak in analytical HPLC (Figure 1A). The oxidative product, in each peptide, was removed during the purification of synthesized analogues. Moreover, the TFA solution in final deprotection resulted to low yield of oxidation. The purification was achieved using semi preparative, reversed-phase HPLC, the peptide purity was assessed by analytical HPLC and by electron spray ionization mass spectrometry (ESI-MS). The purity of the synthesized peptides were verified by analytical HPLC (Figure 1, Figure 2 and Figure 3), the identification and characterization of the final peptides were performed by ESI-MS (Table 1 and Figure 1, Figure 2 and Figure 3).

### 2.2. Inhibitory Activity of MOG APLs in the EAE.

The rat MOG_35–55_ (rMOG_35–55_) peptide (35 μg) induces a chronic disease with a peak clinical score by day 16 after the immunization (Figure 4). Co-injection of linear APL analogues (350 µg of the [Ala^41,46^]MOG_35–55_ or [Ala^41^]MOG_35–55_) with the encephalitogenic peptide MOG_35–55_ (35 µg) at the time of EAE induction (time zero) significantly reduced disease symptoms and incidence, with the linear [Ala^41^]MOG_35–55_ peptide completely preventing the onset of the disease, as compared to PBS (phosphate buffered saline) control (Figure 4). The linear analogue with TyrOMe (methyl ether instead of hydroxyl group) at position 40 [TyrOMe^40^]MOG_35–55_ and the cyclic analogues cyclo(46–55)MOG_35–55_ and cyclo(41–55)MOG_35–55_ did not significantly lower the clinical symptoms of EAE (Figure 4).

This study was an initial attempt to determine which of the designed linear and cyclic modified peptide analogues of human MOG_35–55_ epitope has the greatest efficacy in controlling EAE clinical symptoms. Our decision to use a large quantity of APLs (350 µg, 10-fold excess) in co-injection with the rat MOG_35–55_ epitope was based on our previous studies [10,31] in which the antagonism of MBP_87–99_ modified linear and cyclic peptides in Lewis rats was achieved using more than 15-fold excess.

MOG is a candidate autoantigen in MS and the MOG_35–55_ epitope represents one of the most immunodominant regions in humans and induces chronic EAE in C57BL/6 mice. There is gathering evidence that mutations of disease-associated epitopes (APLs) can actively inhibit disease maybe through modification of interactions with the T cell receptor of encephalitogenic T-cells. Development of novel alternative molecules that mimic the immunomodulatory activity of MOG_35–55_ epitope and maintaining an advantage over regular peptides in terms of stability is a necessary step before these molecules can be used for therapeutic purposes. There are several approaches in the development of such molecules. One is the design and synthesis of non-peptide mimetics with the same biological activity as the parent peptide or with antagonist activity. Another approach is the design of constrained cyclic peptide analogues. The cyclic peptide analogues could be designed using various lengths of bridges for their best flexibility in order to reach and enhance their binding to potential receptors. The selected bridging-atoms should allow for significant side-chain interactions with the receptor in order to be conserved. Cyclic analogues of MOG_35–55_ offer several advantages compared to its linear counterparts such as increased resistance to metabolic degradation and conformational restriction. A potent constrained peptide analogue, offers important structural information regarding its bioactive conformation for further drug design. MOG_35–55_ peptide (based on the rat sequence of MOG with Ser at position 42 instead of Pro in human MOG) induces chronic EAE while the MOG_35–55_ human epitope was used for the design of APLs [32]. The amino acids Arg^41^, Phe^44^, Arg^46^ and Val^47^ of the rat MOG_38–51_ epitope represent primary TCR contact residues while the Gly^38^ and Phe^39^ minor TCR contact sites [37]. Moreover, the development of spontaneous EAE in transgenic mice that express the MOG-specific TCR in the absence of MOG is possible, due to other neurological proteins that share similar TCR contact sites with the immunodominant MOG_35–55_ epitope [38]. The TCR contact sites replacement of either Arg with Ala at positions 41, 46 or Tyr with Tyr(OMe) at position 40, results in linear peptide analogues with inhibitory effects with the [Ala^41^]MOG_35–55_ to present the best inhibition in the EAE model. Furthermore, the cyclic analogues were synthesized by amide bridging the Lys side chain at position 41 or 46 with the C terminal and prevent in different degrees the EAE induced by rat MOG_35–55_. The Arg at position 41 or 46 was substituted by Lys in order its side chain amine group to be used for the cyclization (amide bond) with the carboxyl group in C terminal.

The finding that EAE induced by rat MOG_35–55_ can be ameliorated by co-administration of mutated peptides of human epitope MOG_35–55_ is very intriguing. The lower antagonist activity of cyclic analogue may be attributed to their lower binding ability to major histocompatibility complex (MHC) due to the restricted conformational freedom. Furthermore, lack of agonist activity in the EAE system and expression of antagonist activity by linear analogues, maybe suggests that the guanidino group of Arg at position 41 and 46 are required for recognition by TCR and that the Arg^41^ and Arg^46^ are important residues for EAE induction in combination with the hydroxyl group of Tyr^40^ and Ser at position 42 (rat sequence) instead of Pro (human sequence).

APLs can switch an agonist peptide into an antagonist peptide. Peptides which are involved in the trimolecular complex between the MHC, the antigen (peptide) and the TCR and cause an antagonistic (EAE suppression) effect (ie loss of T cell activation) have loss of main interaction of peptide side chains with the CDR3 loop of the TCR. Mutation of large side chain of the peptide which interact with the TCR to small side chain amino acids (such as Ala) can cause antagonism and thus, inhibit EAE symptoms. Furthermore, APLs can elicit selected effect or functions from T-cells [39,40,41]. In malaria and in autoimmune diseases APLs can be used to switch an epitope-specific Th1-type CD4^+^ T cell response (characterized by production of IFN-γ, IL-2) towards a Th2-type T cell response (IL-4, IL-10, TGFβ) resulting in disease suppression. The recruitment of distinct populations of suppressor T-cells to the autoimmune target, by activation through different TCR, is also possible. APLs may activate regulatory populations of T-cells able to antagonize the action of encephalitogenic cells by limiting their deleterious effect in the CNS. It would, therefore, be very interesting to evaluate *in vitro* the synthesized APLs, in order to explore their immunogenicity, in terms of cell proliferation, antibody and cytokine production.

## 3. Experimental Section

### 3.1. General

All commercially available solvents and reagents were HPLC grade, purchased from Merck (Darmstadt, Germany), Sigma-Aldrich (Saint Louis, MO, USA), Fluka (Buchs, Switzerland) and Fisher Scientific (Loughborough, UK) and they were used without further purification. HPLC-grade water was prepared using a Millipore Simplicity system. 2-chlorotrityl chloride (CLTR-Cl) resin (1% DVB, 200–400 mesh), Fmoc-protected-amino acids and piperidine were purchased from Chemical and Biopharmaceutical Laboratories of Patras (Patras, Greece). Electrospray ionization mass spectrometry (ESI-MS) experiments were performed with a Micromass ZQ Electrospray Platform (Waters Ges.m.b.H., Vienna, Austria) coupled to a MassLynx 4.1 data system (Waters Ges.m.b.H., Vienna, Austria).

### 3.2. Synthesis

#### 3.2.1. Conventional Solid Phase Peptide Synthesis (SPPS) of Linear Peptides

The synthesis of [Ala^41^]MOG_35–55_, [Ala^41,46^]MOG_35–55_ and [TyrOMe^40^]MOG_35–55_ was performed on CLTR-Cl resin (3 g). The first N^a^-9-fluorenylmethyloxycarbonyl (Fmoc) protected amino acid, Fmoc-Lys(Boc)-OH (4.5 equiv.), was esterified to the resin in the presence of diisopropylethyamine (DIEA, 13.5 equiv.) in dichloromethane (DCM, 20 mL) for 1.5 h at RT (room temperature). Subsequently methanol (MeOH, 3 mL) and DIEA (1.5 mL) were added and the mixture stirred for 30 min at RT. The remaining active site of the resin was capped using a mixture of DCM/MeOH/DIEA (85:10:5) at RT. The resin was washed with *N,N*-dimethylformamide (DMF, 3 times × 5 mL) and isopropanol (*i*-PrOH, 3 times × 5 mL) following by the Fmoc deprotection with the 25% piperidine in DMF solution (2 times for 10 min and 30 min) and dried overnight under vacuum. The substitution level, measured by the weight increase, after the first coupling and Fmoc deprotection, was 0.72 mmol/g resin.

The remaining peptide chain was assembled by sequential couplings of the following Fmoc protected amino acids (5.4 equiv.) that were protected in side chain with *tert*-Butyl (tBu), Trityl (Trt) and 2,2,4,6,7-pentamethyldihydrobenzofuran-5-sulfonyl (Pbf): Fmoc-Gly-OH, Fmoc-Asn(Trt)-OH, Fmoc-Arg(Pbf)-OH, Fmoc-Tyr(tBu)-OH, Fmoc-Leu-OH, Fmoc-His(Trt)-OH, Fmoc-Val-OH, Fmoc-Val-OH in the presence of *N,N'*-diisopropylcarbodiimide (DIC) (5.94 equiv.) and 1-hydroxybenzotriazole (HOBt) (8.1 equiv.) in dimethylacetamide (DMAC) for 4–6 h, followed by Fmoc deprotection with piperidine (two times with 25% in DMF for 10 min, and 30 min). The completeness of each coupling and Fmoc deprotection step was verified by the Kaiser test and thin layer chromatography (TLC) using *n*-butanol (1-BuOH)-acetic acid (AcOH)–water (H_2_O) (4:1:1), toluene (Tol)–MeOH–AcOH (7:1.5:1.5) and acetonitrile (ACN)–H_2_O (5:1) as eluent systems. After the couplings of the first nine amino acids, the resin was splitted to three equal amount in separate reactors for the synthesis of the [Ala^41^]MOG_35–55_, [Ala^41,46^]MOG_35–55_ and [TyrOMe^40^]MOG_35–55_ peptides following the described methodology. The following Fmoc protected amino acids were used: (i) [Ala^41^]MOG_35–55_: (1.8 equiv) Fmoc-Arg(Pbf)-OH, Fmoc-Ser(tBu)-OH, Fmoc-Phe-OH, Fmoc-Pro-OH, Fmoc-Pro-OH, Fmoc-Ala-OH, Fmoc-Tyr(tBu)-OH, Fmoc-Trp(Boc)-OH, Fmoc-Gly-OH, Fmoc-Val-OH, Fmoc-Glu(tBu)-OH and Fmoc-Met-OH; (ii) [Ala^41,46^]MOG_35–55_: (1.8 equiv) Fmoc-Ala-OH, Fmoc-Ser(tBu)-OH, Fmoc-Phe-OH, Fmoc-Pro-OH, Fmoc-Pro-OH, Fmoc-Ala-OH, Fmoc-Tyr(tBu)-OH, Fmoc-Trp(Boc)-OH, Fmoc-Gly-OH, Fmoc-Val-OH, Fmoc-Glu(tBu)-OH and Fmoc-Met-OH and (iii) [TyrOMe^40^]MOG_35–55_: (1.8 equiv) Fmoc-Arg(Pbf)-OH, Fmoc-Ser(tBu)-OH, Fmoc-Phe-OH, Fmoc-Pro-OH, Fmoc-Pro-OH, Fmoc-Arg(Pbf)-OH, Fmoc-Tyr(OMe)-OH, Fmoc-Trp(Boc)-OH, Fmoc-Gly-OH, Fmoc-Val-OH, Fmoc-Glu(tBu)-OH and Fmoc-Met-OH.

##### Cleavage from the Resin and Final Deprotection

After completing the synthesis of each peptide, the resin was cleaved with the splitting solution DCM/2,2,2-trifluoroethanol (TFE) (7:3) for 1.5 h at RT. The final deprotection of each peptide was achieved using a trifluoroacetic acid (TFA)/DCM/triethylsilane(TES)/1,2-ethanedithiol(EDT)/anisole/H_2_O (90:4:2:2:1:1) solution for 5 h at RT. The solvent was removed by evaporation and each peptide precipitated with cold diethyl ether. The final amounts of the crude synthesized peptides were: (i) 0.176 g of [Ala^41^]MOG_35–55_; (ii) 0.146 g of [Ala^41,46^]MOG_35–55_ and (iii) 0.1 g of [TyrOMe^40^]MOG_35–55_.

#### 3.2.2. Synthesis of Cyclic Peptides: Cyclo(46–55)MOG_35–55_ and Cyclo(41–55)MOG_35–55_

##### Conventional SPPS of Precursor Protected Linear Peptides

The synthesis of linear protected peptides has been accomplished using the CLTR-Cl (1 g for each peptide) resin and the experimental procedure that was used for the synthesis of linear protected peptides. The next Fmoc protected amino acids (1.8 equiv) were used: Fmoc-Gly-OH, Fmoc-Asn(Trt)-OH, Fmoc-Arg(Pbf)-OH, Fmoc-Tyr(tBu)-OH, Fmoc-Leu-OH, Fmoc-His(Trt)-OH, Fmoc-Val-OH, Fmoc-Val-OH, Fmoc-Arg(Pbf)-OH, Fmoc-Ser(tBu)-OH, Fmoc-Phe-OH, Fmoc-Pro-OH, Fmoc-Pro-OH, Fmoc-Arg(Pbf)-OH, Fmoc-Tyr(tBu)-OH, Fmoc-Trp(Boc)-OH, Fmoc-Gly-OH, Fmoc-Val-OH, Fmoc-Glu(tBu)-OH and Boc-Met-OH. Moreover, the Fmoc protected Lys with the 4-Methyltrityl group [Fmoc-Lys(Mtt)-OH] was used at position 41 and 46 for the synthesis of cyclo(41–55)MOG_35–55_ and cyclo(46–55)MOG_35–55_ respectively.

##### Cleavage from the Resin and Mtt Deprotection

The protected peptides on the resin were treated with the splitting solution DCM/HFIP (6:4) for 1.5 h at RT to remove each peptide from the resin with simultaneous Mtt deprotection from the Lys^41^ and Lys^46^. The solvent was removed by evaporation and each peptide precipitated with cold diethyl ether and dried overnight under vacuum. The final amounts of the crude synthesized peptides were: (i) 0.55 g of protected [Lys^46^]MOG_35–55_ and (ii) 0.5 g of protected [Lys^41^]MOG_35–55_.

##### Cyclization of Protected Peptides and Final Deprotection

To a solution of each of the above linear protected peptides (100 mg, 0.025 mmol) in dry DMF (20 mL), 2,4,6-collidine (0.06 mL, 0.15 mmol) and 1-hydroxy-7-azabenzotriazole (10.21 mg, 0.074 mmol) were added. The solution was then added dropwise to a solution of *O*-benzotriazol-1-yl-*N*,*N*,*N*',*N*'-tetramethyluronium tetrafluoroborate (TBTU) (24.08 mg, 0.075 mmol) in dry DMF (80 mL) for 6 h and the mixture was stirred overnight. The solvent was removed under reduced pressure affording a light-yellow oily residue. The cyclic protected peptide was precipitated from H_2_O and was dried in vacuum for 12 h. The final deprotection of each cyclic peptide was achieved using a trifluoroacetic acid (TFA)/DCM/triethylsilane(TES)/1,2-ethanedithiol(EDT)/anisole/H_2_O (90:4:2:2:1:1) solution for 5 h at RT. The solvent was removed by evaporation and each peptide precipitated with cold diethyl ether. The final amounts of the crude synthesized cyclic peptides were: (i) 0.065 g of cyclo(46–55)MOG_35–55_ and (ii) 0.06 g of cyclo(41–55)MOG_35–55_ (Scheme 2).

### 3.3. Peptide Analysis and Purification

The purity of the synthesized peptides was determined by analytical HPLC (1260 Infinity, Quaternary Pump VL Agilent, Waldbronn, Germany) using an Agilent ZORBAX Eclipse Plus C18 column (3.5 μm, 100 × 4.6 mm, at 214 nm and 254 nm (Table 1). Separation was achieved by gradient elution from 10% ACN to 100% ACN in 30 min at flow rate of 1 mL·min^−1^. The purification of each product was carried out on a semi-preparative RP-HPLC (Waters 600 solvent delivery system, combined with a Waters 996 photodiode array detector, Waters Ges.m.b.H., Vienna, Austria) using a Lichrosorb RP-C18 column (7 μm, 250 × 4 mm, Merck, Darmstadt, Germany). Separation was achieved by gradient elution from 10% ACN to 60% ACN over 45 min at a flow rate of 3 mL/min. The HPLC solvents for the analytical and semi-preparative HPLC were: H_2_O with 0.08% TFA, ACN with 0.08% TFA. The final products were identified by ESI-MS (Table 1). The spectrometric analysis (ESI-MS) was performed using the ion spray source in positive ion mode with the following settings: (i) capillary voltage: 3,5 KV; (ii) cone voltage: 30 V; (iii) source temperature: 140 °C; (iv) desolvation temperature: 250 °C; (v) desolvation gas: 500 L/h; (vi) cone gas: 50 L/h.

### 3.4. Biological Evaluation

#### 3.4.1. Animals and Ethics Statement

In all experimental procedures animals of the species *Mus musculus* were used. C57BL/6 mice were purchased from the Jackson Laboratory (Maine, ME, USA) and were used at 12–18 weeks of age, with a mean body weight of 20 gr. Animal procedures were performed to minimize suffering and conformed to the principles of the three Rs following the guidelines of the EU directive for animal research 2010/63/EU. Ethical permission and experimentation licences were provided by the animal research facility of the Hellenic Pasteur Institute (Athens, Greece)and the General Secretariat of Agricultural Economy (Athens, Greece)and Veterinary Medicine of the Greek State (Athens, Greece) according to the presidential directive 160/91. The reporting of the animal experiments in this study follows the ARRIVE guidelines.

#### 3.4.2. EAE Induction

MOG-EAE was induced in female C57BL/6 (C57 black 6) mice by subcutaneous (s.c.) tail-base injection of 35 µg of rat MOG_35–55_ in 100 μL PBS emulsified in an equal volume of complete Freund’s adjuvant (CFA) supplemented with 400 µg/injection of H37Ra *Mycobacterium tuberculosis* (Difco) [42,43,44]. APLs were added in the same emulsion at 350 µg each and were co-injected at the tail base of mice. Mice also received an intraperitoneal (i.p.) injection of 200 ng of PTx (Sigma-Aldrich) on days 0 and 2 post-immunization. Animals were monitored daily for the clinical signs of EAE according to the following scores: 0, normal; 1, limp tail; 2, hind limb weakness; 3, hind limb paralysis; 4, forelimb paralysis; and 5, moribund or dead (0.5 gradations represent intermediate scores). All mice were allowed free access to food and water throughout the experiments.

## 4. Conclusions

This research has shed light in the design and synthesis of MOG_35–55_ related peptide analogues with crucial TCR mutations with suppressing biological activity in a chronic EAE animal model of MS. This study has shown the importance of Arg at positions 41, 46 and Tyr at position 40 for potency (EAE induction). Utilizing the important feature of cyclization and the presence of Lys^41^ or Lys^46^ for antagonist activity in a linear peptide, potent cyclic antagonist peptides were designed and synthesized. The antagonist potency of linear peptides provided important information for EAE inhibition and will initiate in the future structure-activity relationship studies which will further explore the position 41 and 46 of immunodominant epitope 35–55 of human MOG.
